# A new self-assessment tool following shoulder stabilization surgery, the auto-Walch and auto-Rowe questionnaires

**DOI:** 10.1007/s00167-022-07290-y

**Published:** 2022-12-31

**Authors:** Omar Lazrek, Karam Mark Karam, Pierre-Alban Bouché, Anselme Billaud, Auriane Pourchot, Arnaud Godeneche, Olivier Freaud, Jean Kany, Pierre Métais, Jean-David Werthel, Yoann Bohu, Antoine Gerometta, Alexandre Hardy

**Affiliations:** 1 Clinique Du Sport, 28 Boulevard Saint-Marcel, 75005 Paris, France; 2 Hôpital Lariboisière, 2 Rue Ambroise Paré, 75010 Paris, France; 3 Centre de Chirurgie Orthopédique et Sportive, Mérignac, France; 4 Hôpital Ambroise-Pare, 9 Av. Charles de Gaulle, 92100 Boulogne-Billancourt, France; 5 Centre Orthopédique Paul Santy, Lyon, France; 6 Hôpital Avicenne, Bobigny, France; 7 Clinique de l’Union–Ramsay Santé, Toulouse, France; 8 Hopital Prive de la Châtaigneraie–ELSAN, Clermont-Ferrand, France

**Keywords:** Shoulder, Postoperative, Patient reported outcome, Instability

## Abstract

**Purpose:**

Patient-reported outcome measures (PROMS) are increasingly used for patient evaluation, as well as for scientific research. Few are used for practical purposes in the clinical setting, and few are reliable enough to allow proper feedback to physicians. Two of the most commonly used assessment tools in shoulder instability are the Walch–Duplay and the Rowe scores. The aim of this study was to evaluate the validity of self-administered versions of the Walch–Duplay and Rowe scores following shoulder stabilization procedure.

**Methods:**

Between the months of May and December 2021, all patients who were followed in one of six institutions for shoulder instability were included. Patients were required to anonymously fill a self-administered version of Walch–Duplay and Rowe score. The classic scores were measured by the surgeon. Correlations between self-assessment and physician-assessment were then recorded.

**Results:**

A total of 106 patients were evaluated during the study period. Using the Spearman coefficient for correlation, a strong correlation (*r* > 0.5) was found between the results of the self-administered questionnaire and the surgeon-measured score. The difference between surgeon- and patient-administered questionnaires was non-significant.

**Conclusion:**

The self-administered version of the Walch–Duplay and Rowe questionnaires can reliably be used in the clinical setting for post-operative follow-up of patients undergoing shoulder stabilization procedures.

**Level of evidence:**

Level II.

## Introduction

Recently, interest has been growing to evaluate the capacity to convert historical shoulder scores to patient-reported outcome measures (PROMs). Among the shoulder scores, studies show that the ASES shoulder score, the Oxford shoulder score and the VAS have successfully been converted into PROMs [1]. The DASH score has also been found to be useful and reproducible for the evaluation of rotator cuff pathology as a PROM [2–4]. Chelli et al. found that the Constant–Murley score, although associated with some discrepancies between sections, could also accurately be estimated by self-administration by patients in general shoulder pathology [5].

Shoulder instability, as well as its treatment is evaluated by several scores, such as the Rowe and Walch–Duplay scores. The Rowe score is one of the most frequently used assessment tools according to a 2010 study by Rouleau et al. [6], as well as a systematic review by Fanning et al. [7]. One of the most widely used scores in Europe is the Walch–Duplay score, recommended for use by the European Society of Shoulder and Elbow Surgery [8]. It correlates well with the subjective questionnaire of the Western Ontario Shoulder instability Index, which is a PROM [9]. Such scores are usually calculated following a clinical examination. The clinical relevance of those scores has been well demonstrated in the literature [6, 10]; however, their use remains limited because of their clinician-dependent quality [1].

Although the effectiveness of such scores as an assessment tool are well established, they are time consuming in the clinical setting, as no self-assessment has been developed using the aforementioned parameters. The main objective of this study was to evaluate the comparability and correlation of patient-reported version of Walch–Duplay and Rowe questionnaires and assess their reliability compared with a standard score calculated by a senior surgeon.

The hypothesis was that the Walch–Duplay and Rowe questionnaires could become PROMs by use of an adapted self-administered questionnaire, which would be useful in the post-operative setting, by speeding up and streamlining the follow-up process as well as providing an easy tool for patients to obtain more concrete information about their progress and optimize involvement in their own care.

## Materials and methods

After approval by the Institutional Review Board of the Clinique du Sport in Paris (IRB00010835), a multicentric continuous prospective cohort study was undertaken, evaluating patients seen in the office between the months of May and December 2021, regardless of timing after surgery. The patients had previously undergone shoulder stabilization surgery (arthroscopic Bankart repair vs. open or arthroscopic Latarjet procedure) based on the ISIS score, as previously described by Thomazeau et al. [11]. Two questionnaires were created, one adapted to the items of the Rowe score as shown in Table 1, and another to the items of the Walch–Duplay score, as shown in Table 2, and, after obtaining informed, written consent, given to all patients seen in the clinic during the study period. The Rowe score has undergone modifications [12], but the original one contains sections for function (50 points), mobility (20 points) and stability (30 points). The Walch–Duplay score is divided into 4 main sections with 25 points attributed to each. They are sport or daily activity, pain, stability, and mobility. The original Rowe score was chosen rather than its later editions as reference to create the auto-Rowe questionnaire [12, 13]. The Rowe and Walch–Duplay scores were then calculated by a senior surgeon who was blinded to the result of the self-assessment during the examination. A comparison of the answers of both patient and surgeon were compared. The main outcome was correlation between surgeon and patient-administered questionnaire scores.

**Table 1:** Details of the Rowe score

Rowe score	
**Function**	**(/50 points)**
No limitation in work and sports	30
No limitation in work, mild limitation in sports	25
Mild limitation in work above head and sports	10
Marked limitation and pain	0
**Stability**	**(/30 points)**
No recurrence, subluxation, or apprehension	50
Apprehension when placing arm in certain positions	30
Subluxation (not requiring reduction)	10
Apprehension test positive or notion of instability	0
**Mobility**	(/20 points)*
Normal mobility in ER, IR and elevation	20
75% of ER, IR and elevation	15
50% loss of normal ER, 75% of IR, and elevation	5
No ER, 50% of IR, and elevation	0
**Total**	**(/100 points)**
Excellent	90–100 pts
Good	75–89 pts
Average	51–74 pts
Bad	< 50 pts

*ER external rotation, and *IR* internal rotation

**Table 2:** Details of the Walch–Duplay score

Walch–Duplay score		
**Sport**	**(/25 points)**	**Daily activity (if no sport practiced)**
Return to same sport, at the same level	+ 25	No discomfort
Back to same sport, but at a decreased level	+ 15	Slight discomfort in forceful movements
Change in sport	+ 10	Slight discomfort during simple movements
Stop sport	0	Severe discomfort
**Stability**	**(/25 points)**	
No apprehension	+ 25	
Persistent apprehension	+ 15	
Feeling of instability	0	
True recurrence of subluxation or dislocation	− 25	
**Pain**	**(/25 points)**	
No pain or pain during certain climatic conditions	+ 25	
Pain during forceful movements or when tired	+ 15	
Pain during daily life	0	
**Mobility**	(/25 points)*	
Pure frontal abduction against a wall: symmetrical	+ 25	
Limitation of IR < 3 vertebrae		
Limitation of ER2 to < 10% of the contralateral side		
Pure frontal abduction against a wall < 150°	+ 15	
Limitation of IR < 3 vertebrae		
Limitation of ER2 to < 30% of the contralateral side		
Pure frontal abduction against a wall < 120°	+ 5	
Limitation of IR < 6 vertebrae		
Limitation of ER2 to < 50% of the contralateral side		
Pure frontal abduction against a wall < 90° 0		
Limitation of IR > 6 vertebrae		
Limitation of ER2 to < 50% of the contralateral side		
**Total**	**(/100 points)**	
Excellent	91–100 points	
Good	76–90 points	
Fair	51–75 points	
Poor	< 50 points	

*ER external rotation, *IR* internal rotation, and *ER2* external rotation in abduction

### Inclusion criteria

All patients seen in one of six institutions post-operatively, following a shoulder stabilization procedure, and who were able to read were selected. The questionnaires were administered irrespective of post-operative delay. All surgeries were performed by senior surgeons with sports and/or shoulder specialty training. The types of surgeries performed were arthroscopic Bankart procedures and open or arthroscopic Latarjet procedures. The Latarjet technique used depended on the site and on the operating surgeon.

### Exclusion criteria

All patients below the age of 18 or refusing to participate in the study were excluded.

### Questionnaire

All 106 patients were given both questionnaires to fill upon arrival, before their clinical examination. The questionnaires were designed in order not to require any assistance to be completed. They were composed of questions with a selection of answers in checkboxes as well as instructions for self-evaluation of range of motion, paired with explanatory illustrations detailing the varying ranges per motion type.

**Fig. 1:**
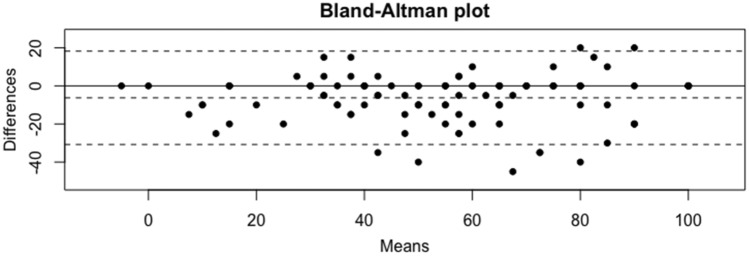
The “Auto” Walch–Duplay score-adapted questionnaire administered post-operatively to patients prior to examination with their surgeon

**Fig. 2:**
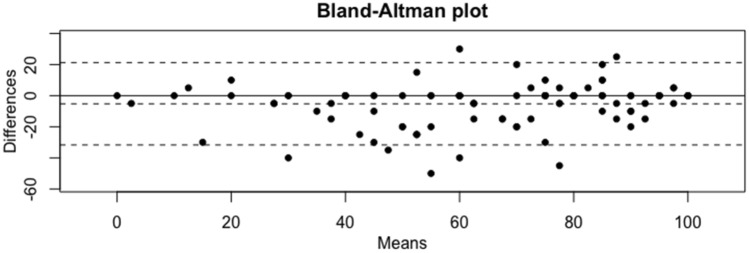
The “Auto” Rowe score-adapted questionnaire administered post-operatively to patients prior to examination with their surgeon

The questionnaires, including the visual aids used to assess the patients, are shown in Figs. 1, 2 with the scores attributed for each section. The points’ distribution for each score is shown in Tables 1, 2.

### Surgeon:

After filling out their questionnaires, all patients were subsequently examined by a senior shoulder surgeon. No prior instruction or information was given regarding the result. The patient was examined with the use of a goniometer to accurately assess range of motion, first on the unaffected healthy shoulder, then on the operated side. The data were collected separately for the Walch and “auto-Walch” scores, and for the Rowe and “auto-Rowe” scores.

### Test–retest reliability

To assess validity of the questionnaires employed, a test–retest protocol was employed. In order for this step to be reliable, there needs to be a sufficient time interval between both questionnaire administrations per patient to avoid recollection. The questionnaires of this study were administered a minimum of 3 days apart and the data were recollected.

### Statistical analysis

A certified statistician performed the statistical analysis. To ensure the study strength, a sample size of 100 patients was found to produce a two-sided 95% confidence interval inferior to 0.20 when the estimate of Spearman’s rank correlation is above 0.75 to increase precision. A 5% non-response rate was expected, and so, the population size was increased to 105 patients at least. There were 106 patients in this study, corresponding to the population of patients recruited by the different centers during the study period. Continuous quantitative variables were described by the mean and standard deviation (SD). Dichotomous variables were described by their number of events and their percentage. Correlations between surgeon and patient score were estimated using Spearman coefficients. The correlation was considered to be «strong» (*r* > 0.5), «moderate» (0.5 < *r* < 0.3) or «weak» (0.3 < *r* < 0.1). Correlation was measured for the full questionnaires as well as individual sections pertaining to the respective sections of the Walch–Duplay and Rowe scores. All tests were 2-sided. The R software (version 3.5.0) was used to perform the statistical analyses. Differences between surgeon and patient scores were calculated with positive differences signifying an underestimation of the score by the patient, and negative differences expressing an overestimation, compared to the surgeon’s examination.

## Results

A total of 106 patients were evaluated during the study period. There were 72 (67.9%) men. Eighty-eight (83%) patients were right-handed and 55 (52.4%) patients were evaluated for their right side. Sixty-one patients (58%) were evaluated for their dominant side. A spearman correlation coefficient was calculated for each section of the individual scores, as well as an analysis of difference between surgeon and patient. The results notably show no significant difference between groups except when evaluating for the mobility item of both questionnaires.

### Walch–Duplay score

There was a strong correlation between surgeon- and patient-reported scores. The average overall score for surgeon- and patient-calculated Walch–Duplay scores were 59.1(25.38) and 52.8(25.98), respectively. There was a high correlation between patient- and surgeon-reported scores, with the lowest spearman coefficient *R* = 0.66, associated with the stability item. However, the difference between scores was not found to be significant (n.s.). Patients scored higher than their surgeons when evaluating their internal rotation (*p* = 0.03). They scored lower, however, in scoring their pain (*p* = 0.03), and their mobility (*p* = 0.02). The highest correlation according to the spearman coefficient was found for the overall Walch–Duplay score, with no significant difference (*p* = 0.08).

### Rowe score

There was also a strong correlation between surgeon- and patient-reported scores. The average overall score was 68.5 and 63.3, respectively, for surgeons and patients. The final score had the second highest spearman coefficient at 0.85. The highest correlation was found in the function item (*R* = 0.88). The lowest correlation was found in the Abduction item (*R* = 0.67) with no significant difference. As was the case for the Walch–Duplay score, significant differences were found in the mobility and internal rotation items of the Rowe score. With the different scoring method for internal rotation, it was found that patients scored lower than their surgeons. They also generally scored their mobility lower.

**Fig. 3:**
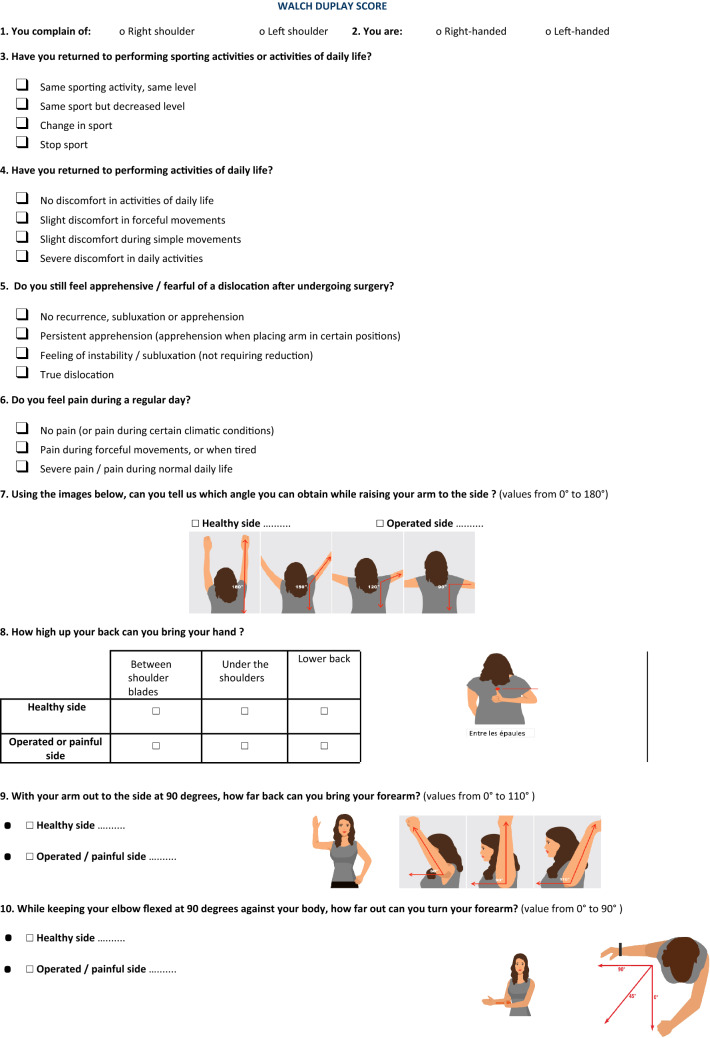
Bland–Altman Plot for Walch–Duplay score. Comparison of the differences between surgeon- and patient- reported score. There is a high level of accuracy between both methods when the plot points fall within the dotted lines, which correspond to the 95% confidence interval

**Fig. 4:**
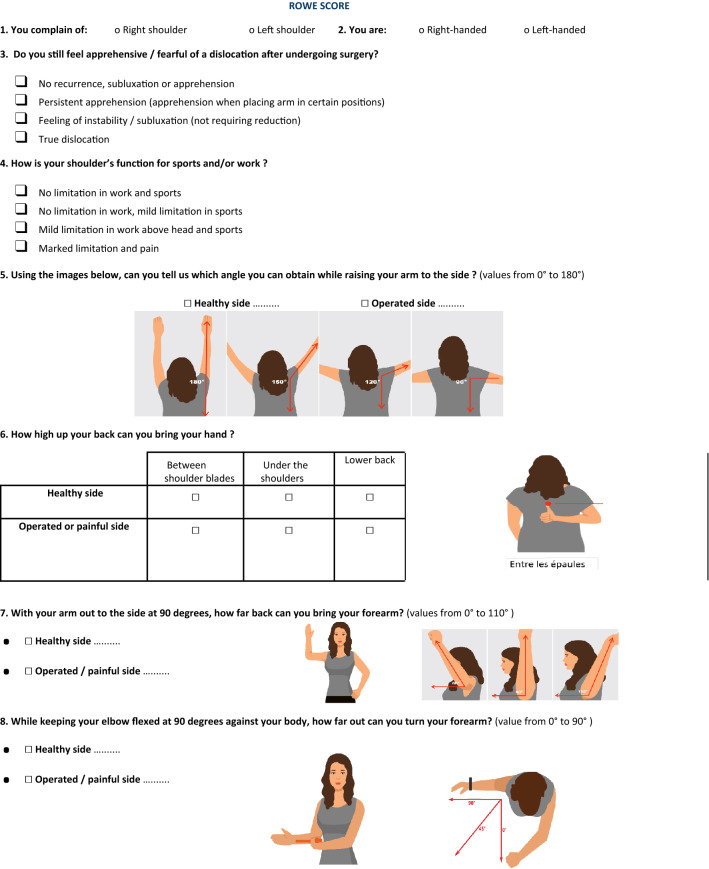
Bland–Altman Plot for Rowe score. Comparison of the differences between surgeon- and patient-reported score. There is a high level of accuracy between both methods when the plot points fall within the dotted lines, which correspond to the 95% confidence interval

The results are detailed in Table 3. The analysis of difference is shown in Figs. 3 and 4.

**Table 3:** Walch–Duplay and Rowe scores as reported by patients and surgeons, with analysis of correlation and difference

	Patient	Surgeon	Spearman	Difference	*p*.value
	(*n* = 106)	(*n* = 106)	[CI 95%]	Surgeon–patient	
Walch–Duplay score					
Return to activity	10.9 (9.5)	10.9 (9.4)	0.9 [0.76; 0.93]	− 0.1 (4.7)	(n.s.)
Stability	17.9 (11.2)	19.8 (10.2)	0.7 [0.51; 0.79]	1.9 (6.5)	(n.s.)
Pain	16.3 (7.8)	18.2 (8.3)	0.8 [0.67; 0.82]	1.8 (6.1)	0.03
Abduction	156.8 (34.2)	161.6 (32.7)	0.7 [0.50; 0.80]	4.8 (21.9)	(n.s.)
Internal rotation	2.1 (0.8)	1.9 (0.8)	0.7 [0.57; 0.80]	− 0.2 (0.6)	0.03
External rotation 2	78.4 (30.4)	76.7 (26.1)	0.8 [0.64; 0.83]	− 1.7 (20.7)	(n.s.)
Functional score	45.1 (21.1)	48.8 (20.4)	0.9 [0.80; 0.93]	3.7 (8.7)	(n.s.)
Mobility score	7.6 (9.7)	10.2 (10.3)	0.7 [0.58; 0.80]	2.6 (7.7)	0.02
Final score	52.8 (26.0)	59.1 (25.4)	0.9 [0.81; 0.92]	6.3 (12.5)	(n.s.)
Rowe score					
Stability	38.2 (15.0)	40.9 (14.2)	0.7 [0.56; 0.83]	2.7 (9.4)	(n.s.)
Function	18.9 (10.9)	19.6 (10.2)	0.9 [0.81; 0.93]	0.7 (5.5)	(n.s.)
Abduction	156.8 (34.2)	161.6 (32.7)	0.7 [0.51; 0.79]	4.8 (21.9)	(n.s.)
Internal rotation	2.1 (0.8)	1.9 (0.8)	0.7 [0.54; 0.79]	58.8 (25.6)	< 0.01
External rotation 1	61.0 (27.5)	60.9 (25.3)	0.7 [0.58; 0.84]	− 0.2 (18.1)	(n.s.)
Score without mobility	57.1 (23.3)	60.5 (21.0)	0.9 [0.78; 0.91]	3.4 (11.4)	(n.s.)
Mobility score	6.2 (7.3)	8.0 (7.4)	0.8 [0.62; 0.83]	1.8 (5.4)	0.04
Final score	63.3 (26.7)	68.5 (24.2)	0.9 [0.75; 0.91]	5.2 (13.5)	(n.s.)

### Questionnaire validity

Test–retest reliability was assessed among 11 patients. The time interval was 3.91 days on average. An intra-class correlation coefficient for the final Walch–Duplay score was 0.98 with a confidence interval of 95% [0.94; 0.99]. Similarly, the final Rowe score had an ICC of 0.98 [0.93; 0.99]. This translates to an “excellent” result.

## Discussion

The main result of this study was found to be the absence of difference between the overall self-reported and surgeon-reported Walch–Duplay and Rowe scores. Statistically significant difference was observed within sections, notably the internal rotation and mobility items of both scores, as well as the pain item of the Walch–Duplay score. In both the Walch–Duplay and Rowe questionnaires, patients significantly underestimated their overall mobility compared to the surgeon assessments. Patients scoring their pain and mobility scores lower are telling. They may be explained by the lack of measurement tools that were made available to patients during self-evaluation; they may also be explained by a surgeon’s desire to highlight post-operative improvement and positive results by “embellishment”.

The secondary result was the overall strong correlation (Spearman > 0.5) between self-reported and surgeon-reported Walch–Duplay and Rowe scores. The lowest Spearman coefficient for the Walch–Duplay score was 0.66. The lowest Spearman coefficient for the Rowe score was 0.67. This shows agreement within all items of both scores. In a systematic review, Pattabhiraman et al. found that there was a high level of agreement between patients and clinicians in most categories, but that the rotation component in some scores could be improved [14]. This had previously been observed in a prospective study by Rüdiger et al. to study the correlation between patient and surgeon assessments of mobility [15]. This could explain the significant difference we found in mobility in both Rowe and Walch–Duplay scores.

Precedent exists for converting shoulder scores to become completely patient-assessed. Usually, this is done using visual aids. Levy et al. have converted the Constant–Murley score in English, and Chelli et al. used a French version with good agreement between patients and clinicians [5, 16]. This has been done for other scores as well, such as the ASES score, the SF-36, and the SPADI scores [5, 17–20]. To our knowledge, this is the first attempt at converting the Rowe and Walch scores into self-assessment questionnaires. The need and interest for such tools is growing as it allows surgeons to be freed from sometimes tedious data entry while involving the patients further in their own health. It is therefore important to not lose the quality and clinical relevance of a standardized and validated measure, and one must know when those can be applied [21, 22].

Development of these tools also promotes and facilitates communication between specialists within and between institutions, and advances the scientific knowledge. The research committee of the American Shoulder and Elbow Surgeons demonstrated this in 1994 [23].

Full compliance was obtained in this study. This may be due to the simplicity of the adapted self-administered questionnaire as well as the opportunity patients received to glimpse into the way surgeons specifically assess their own work and, therefore, their patient’s post-operative status/result.

The minimum (0) and maximum (100) values were not reached for the scores filled by either patient or surgeon. Therefore, there was no ceiling or floor effect. According to Terwee et al. [24], in the presence of a ceiling or floor effect of more than 15%, there is an inherent problem with the validity of the contents when generating questionnaire items.

The development of an Auto-Walch/Auto-Rowe questionnaire provides several benefits. First of all, it gives patients further implication in their own care and follow-up, and provides potential concrete information which may work as a motivator in the recovery process between follow-ups. It also frees surgeons and clinicians from tedious repetitive work while still providing data for potential clinical scientific studies to be performed. Finally, it enables patients to have increased confidence in their post-operative function. Discovering that their performance is generally underestimated could boost provide a psychological boost.

In seeking to validate the assessment tools, a test–retest process was undertaken. An average interval of 3.91 days was employed. This is a slightly shorter time than some studies in the literature [18]; however, this depends on the tool being assessed and validated [25]. As a matter of fact, some studies do not show that a duration less than 7 days significantly affects the results [26]. This interval was deemed appropriate for the condition being studied. Our questionnaires were considered to have good validity.

There are some limitations to this study. First of all, there was no post-operative delay defined, which means that some patients were evaluated at their first post-operative visit, and some were seen at final follow-up. This explains the low mean Walch–Duplay and Rowe scores obtained, and the low amount of ceiling effect. However, the high degree of correlation between self- and surgeon-administered questionnaires at different post-operative moments shows that the Auto-Walch/Auto-Rowe tool is valid for many time points. Furthermore, patients having undergone different types shoulder stabilization procedures were seen, also potentially affecting the result, especially in the acute setting. Although this is the case, it can be deduced that these questionnaires can be used in the post-operative setting of different shoulder stabilization procedures.

In the future, it would be interesting to learn which factors negatively affect patients’ self-perception of their abilities leading to underestimation of performance in range of motion. This would help speed up recovery, and allow surgeons to select which patients have actually improved more than previously estimated. Another point to study in the future would be the correlations between self-administered and surgeon-administered questionnaires with respect to type of procedure and timing of surgery, which would allow confirmation of questionnaire validity at all post-operative follow-ups.

## Conclusion

This study shows that the Walch and Rowe questionnaires can be easily converted to self-administered questionnaires without losing the quality of the original index. A high correlation between surgeon- and patient-administered questionnaires was observed, along with non-significant differences between overall scores.

## Data Availability

The data of this study can be made available upon request to the corresponding author.
